# Identification of *Mycobacterium tuberculosis* Antigens with Vaccine Potential Using a Machine Learning-Based Reverse Vaccinology Approach

**DOI:** 10.3390/vaccines9101098

**Published:** 2021-09-28

**Authors:** Blaine Teahan, Edison Ong, Zhenhua Yang

**Affiliations:** 1 Epidemiology Department, School of Public Health, University of Michigan, Ann Arbor, MI 48109, USA; bteahan@umich.edu; 2 Department of Computational Medicine and Bioinformatics, University of Michigan, Ann Arbor, MI 48109, USA; edong@umich.edu

**Keywords:** tuberculosis, vaccines, protective antigens, machine learning, reverse vaccinology

## Abstract

Tuberculosis (TB) is the leading cause of death of any single infectious agent, having led to 1.4 million deaths in 2019 alone. Moreover, an estimated one-quarter of the global population is latently infected with *Mycobacterium tuberculosis* (MTB), presenting a huge pool of potential future disease. Nonetheless, the only currently licensed TB vaccine fails to prevent the activation of latent TB infections (LTBI). These facts together illustrate the desperate need for a more effective TB vaccine strategy that can prevent both primary infection and the activation of LTBI. In this study, we employed a machine learning-based reverse vaccinology approach to predict the likelihood that each protein within the proteome of MTB laboratory reference strain H37Rv would be a protective antigen (PAg). The proteins predicted most likely to be a PAg were assessed for their belonging to a protein family of previously established PAgs, the relevance of their biological processes to MTB virulence and latency, and finally the immunogenic potential that they may provide in terms of the number of promiscuous epitopes within each. This study led to the identification of 16 proteins with the greatest vaccine potential for further in vitro and in vivo studies. It also demonstrates the value of computational methods in vaccine development.

## 1. Introduction

Despite being an ancient disease, tuberculosis (TB) persists as one of the ten leading causes of death globally and the leading cause of death of any single infectious agent with 10 million new cases and 1.4 million deaths in 2019 alone [1,2]. The emergence of HIV and multidrug-resistant TB in the past several decades has further complicated global TB control [3,4].

In light of the significant impact of TB on global health, the World Health Organization (WHO) has prioritized the control of the TB epidemic, launching the End TB Strategy, which aspires to reduce the number of TB deaths by 95% and TB incidence by 90% by 2035, as compared to the 2015 figures [5,6]. Despite this ambition, the current pace of progress suggests that neither goal will be met [7]. Compounding the inadequacy of the current progress, the coronavirus disease 2019 (COVID-19) pandemic has impaired TB surveillance, which could increase TB mortality by 13%, undoing the last five years’ progress [7].

Perhaps the greatest barrier to meeting these goals is the absence of a universally effective vaccine against all types of TB. This is in part because the unique natural history of MTB infection makes it a difficult vaccine target. MTB is able to survive many years within host immune cells, suppressing the intracellular attacks of the macrophage [8]. In order to contain these persistent bacteria, the host forms a granuloma—an aggregate of host immune cells encasing the site of infection [9,10]. This state of persistent asymptomatic infection is referred to as latent TB infection (LTBI). If the host immune system can no longer contain the bacteria, LTBI may activate to post-primary TB, including highly contagious pulmonary TB, which accounts for a majority of TB cases and deaths [1,11]. The WHO estimates that one-quarter of the global population has an LTBI, representing a huge pool of potential future disease [1]. Unfortunately, there are currently no licensed vaccines that prevent the activation of LTBI. Despite its efficacy in preventing miliary and meningeal TB in infants, the only licensed TB vaccine, Bacillus Calmette-Guérin (BCG), which is derived from an attenuated strain of *Mycobacterium bovis*, has widely variable efficacy against pulmonary TB in adult populations, ranging from 0 to 80%, being least efficacious in tropical climates, which is perhaps due to immunological sensitization to environmental mycobacteria [12,13]. Thus, in order to End TB by 2035, a new vaccine strategy that prevents both primary and post-primary TB in all age groups must be developed.

One promising approach that may compensate for the inadequacies of BCG in sensitized populations is the use of subunit vaccines given in conjunction or succession with BCG [12]. The first attempt at this strategy was the development of MVA85A [14]. Despite great promise, subsequent clinical trials showed that it was no more effective than BCG alone [15]. The candidate vaccine M72 has shown more success, offering 54% protection against pulmonary TB [16]; however, the need for an even more protective vaccine persists.

In light of the observation that MTB modifies the expression of many genes in the hypoxic and nutrient-starved conditions of the granuloma, more recent efforts have employed a subunit vaccine strategy that includes antigens relevant to both TB virulence and latency [17]. It has been hypothesized that such a vaccine can prevent both primary and post-primary TB [17]. Examples of such multistage vaccines include H56, which in early clinical trials has demonstrated better containment of late-stage TB than BCG alone, and ID93, which has been shown in animal models to protect against TB and in Phase I trials has elicited humoral and cell-mediated responses in humans [17,18,19,20]. The protective efficacy of these vaccines remains to be seen in later-stage clinical trials.

Despite the encouraging progress made with these recent candidate vaccines, it is unlikely that a single vaccine will be protective against all forms of TB in all age groups, and so diversification of candidate vaccines is important [21]. Thus, a broader search for MTB protective antigens (PAgs) is anticipated to provide a variety of new candidates and inform the design of new and effective multistage TB vaccines. While previous studies have applied bioinformatic strategies to the selection of PAgs for vaccine candidates, machine learning (ML) has not been previously used for this task. Additionally, reverse vaccinology (RV) allows for the identification of PAgs that may otherwise be impossible to identify or isolate using conventional methods [22]. Furthermore, while traditional RV methods primarily consider surface-exposed proteins, ML-based RV methods allow for the identification of non-surface-exposed proteins. This may be especially important for the development of vaccines against intracellular pathogens, such as MTB, because these non-surface-exposed proteins may induce cell-mediated immunity, which is critical to the control and clearance of intracellular infection. To contribute to the growing knowledge of PAgs in MTB, we conducted the present study employing a recently developed ML-based RV model, Vaxign-ML [23].

## 2. Materials and Methods

Vaxign-ML was applied to the MTB H37Rv proteome (Uniprot Proteome UP000001584) to compute the protegenicity score of each protein [24]. This score predicts the likelihood that a given protein will be a PAg. As described previously by Ong et al., Vaxign-ML used 397 bacterial PAgs with at least one experimental evidence of protection (e.g., in an animal challenge assay) to train an extreme gradient boosting model [23]. With a recommended protegenicity score threshold, Vaxign-ML achieved the highest performance with 0.96 weighted F1-score in a nested five-fold cross-validation and outperformed other existing web-based RV tools [23,25].

We then selected proteins with the previously recommended Vaxign-ML protegenicity score threshold [23]. Of these proteins, those that had previously been established as PAgs according to Protegen were excluded from selection so that all selected prospective PAgs were novel. Protegen is a web-based database that compiles PAgs of several pathogens, including MTB, which are curated from peer-reviewed articles [26]. The remaining proteins were designated as novel Vaxign-ML-predicted PAgs. This list of predicted PAgs was further refined using two independent criteria.

The first criterion considered whether each novel Vaxign-ML-predicted PAg belonged to the family of an established MTB PAg. The rationale for this selection criterion was that novel Vaxign-ML-predicted PAgs that belong to the protein family of an established PAg were likely to be similar in structure, function, and amino acid sequence to the established PAgs and thus were more likely to be PAgs themselves.

To do this, we compiled a list of the protein families of the established MTB PAgs using Protegen and the UniProt Knowledgebase, which is a manually annotated database of protein sequence data combined with summaries of experimentally verified or computationally predicted functional information about each protein [27]. The protein family for each novel Vaxign-ML-predicted PAg for which it was available was also identified using the UniProt Knowledgebase. Novel Vaxign-ML-predicted PAgs that belonged to the protein family of an established PAg were selected.

The second criterion considered whether each novel Vaxign-ML-predicted PAg was involved in biological processes related to either MTB virulence or LTBI. The rationale for the selection of proteins involved in MTB virulence was that PAgs are likely to come from virulence factors. The rationale for the selection of latency-related proteins was their importance in inducing a cell-mediated immune response in the latency of TB. As it has been hypothesized, we were operating under the assumption that a more effective vaccine preventing activation of LTBI could be developed by combining PAgs involved in virulence with those expressed in the latent stage of the disease [17]. 

The Gene Ontology (GO) biological processes of each novel Vaxign-ML-predicted PAg for which they were available were gathered via the UniProt Knowledgebase [27]. Gene Ontology is a project to systematically categorize the function of proteins in terms of molecular function, cellular component, and biological process. We then selected by literature review eleven categories of GO biological processes that were relevant to the unique pathophysiology of MTB in either latency or virulence, including cell envelope biogenesis and maintenance [28,29], DNA repair [30], interaction with host immune system [8], fatty acid beta-oxidation [31], growth in host [32], protein folding [33], response to antibiotic [34], response to acidic pH [35], response to hypoxia [36], response to nitrosative or oxidative stress [32], and response to starvation [37]. Novel Vaxign-ML-predicted PAgs whose biological processes belonged to at least one of these categories were selected.

Upon first selecting the proteins with sufficiently high Vaxign-ML protegenicity scores and then refining this list using the two selection criteria mentioned above, we finally selected the proteins with the greatest number of promiscuous MHC-I and MHC-II epitopes using T-cell epitope prediction. The rationale for this final selection was the necessity that vaccine candidates provide broad population coverage. The presence of promiscuous epitopes is important in the prediction of T-cell epitope candidates because of the highly polymorphic nature of HLA alleles [38]. 

For MHC-I binding prediction, the IEDB-recommended NetMHCpan-4.1 prediction method was used. This method employs an ML strategy trained on both binding affinity and mass spectrometry-eluted ligands [39,40]. Binding predictions were made for 9-mer epitopes with a reference set of 27 frequently occurring HLA alleles which together cover >97% of the global population [41]. The selection threshold for MHC-I binding prediction was percentile rankings less than 1%, as has previously been recommended [42,43].

For MHC-II binding prediction, the IEDB-recommended prediction method was used [40]. This method employs the consensus approach, which combines the NN-align, SMM-align, CombLib, and Sturniolo methods if a corresponding predictor is available for the given molecule, and if not, NetMHCIIpan is used [38,39,44,45,46,47]. Binding predictions were made for 15-mer epitopes with a reference set of 27 frequently occurring HLA alleles, which together cover >99% of the global population [48]. The selection threshold for MHC-II binding prediction was adjusted percentile rankings less than 10%, as has previously been recommended [49]. 

For both MHC-I and MHC-II binding prediction, epitopes that were predicted to bind with high affinity (i.e., binding percentile rank meeting the threshold) to at least four different HLA alleles within the reference set were considered promiscuous epitopes.

The novel Vaxign-ML-predicted PAgs having been selected by the two criteria mentioned above were ranked by the number of promiscuous epitopes they contained for both MHC-I and MHC-II separately. The top-ranked 11 proteins in each ranking were selected.

## 3. Results

### 3.1. Novel Protective Antigens Predicted by Vaxign-ML 

**Figure 1:**
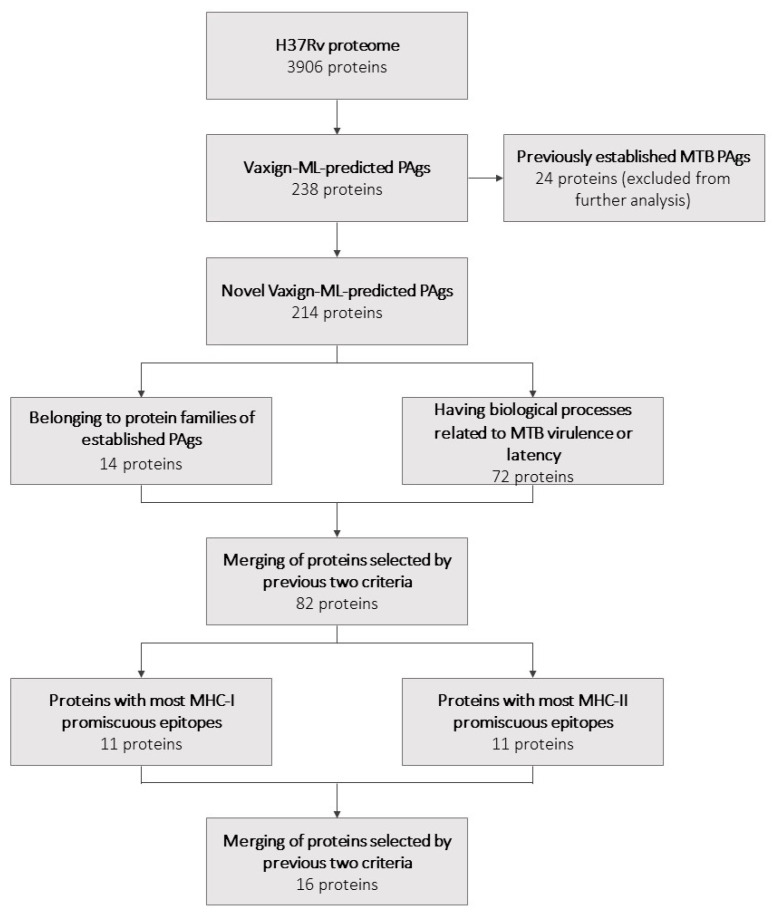
A flowchart summarizing the selection of potential vaccine candidates and the number of proteins selected at each step.

Vaxign-ML was used to predict the protegenicity of the entire H37Rv proteome. This step yielded 238 proteins with protegenicity scores meeting the recommended threshold. Of these 238 proteins, 24 had previously been established as PAgs according to Protegen and thus were excluded from further selection. The remaining 214 novel Vaxign-ML-predicted PAgs (Figure 1, Table S1) were subjected to further selection as described in the following sections.

### 3.2. Antigens Belonging to Protein Families of Previously Established MTB PAgs

A list of all previously established PAgs was gathered using Protegen (Table S2). The protein family of each for which it was available was determined using UniProt. These established PAgs belonged to 11 unique protein families (Table S2). The protein family of each novel Vaxign-ML-predicted PAg for which it was available was also determined using UniProt. The 214 novel Vaxign-ML-predicted PAgs belonged to 116 unique protein families (Table S1). There were 14 novel Vaxign-ML-predicted PAgs that together belonged to seven unique protein families of established PAgs (Figure 1; Table 1).

### 3.3. Antigens Having Biological Processes Associated with MTB Virulence or LTBI

**Figure 2:**
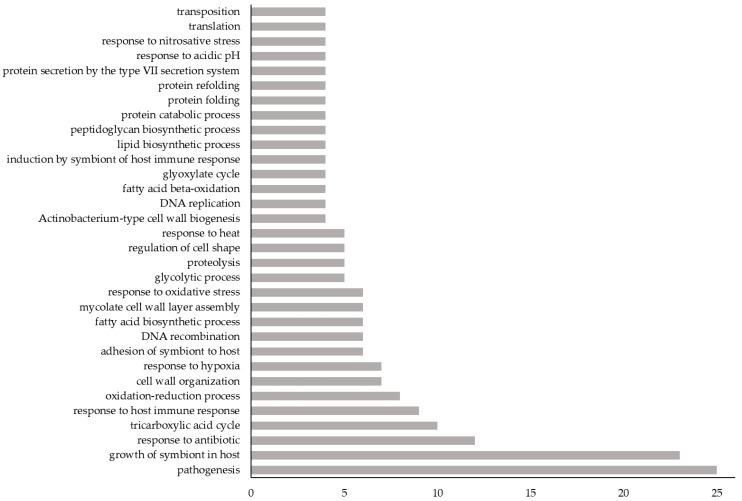
The frequency of GO biological processes among the 214 novel Vaxign-ML-predicted PAgs. *X*-axis: number of novel Vaxign-ML-predicted PAgs having the given GO biological process; *Y*-axis: GO biological processes to which at least 4 novel Vaxign-ML-predicted PAgs belonged. For a complete list of GO biological processes belonging to these 214 proteins, see Table S1.

The GO biological processes of each novel Vaxign-ML-predicted PAg for which they were available were identified using UniProt. These 214 proteins were involved in 226 unique biological processes, of which the most common were pathogenesis and growth of symbiont in host (Figure 2).

**Figure 3:**
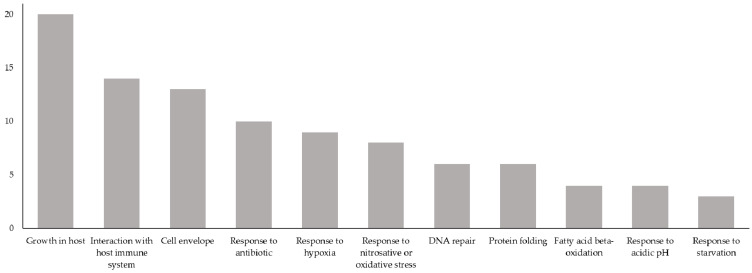
Biological process categories of the 72 proteins having biological processes related to the virulence or latency of MTB. *X*-axis: biological process categories relevant to MTB virulence or latency; *Y*-axis: number of novel Vaxign-ML-predicted PAgs having a GO biological process belonging to a given biological process category. Total exceeds 72 because several novel Vaxign-ML-predicted PAgs have GO biological processes belonging to more than one biological process category.

In summary, there were 72 novel Vaxign-ML-predicted PAgs that had GO biological processes that belonged to one or more of these biological process categories (Figure 1, Table 2). The 72 proteins selected here had biological processes that most commonly belonged to the growth in host, interaction with the host immune system, and cell envelope biogenesis and maintenance categories (Figure 3).

### 3.4. Antigens with the Greatest Number of Promiscuous MHC-I and MHC-II Epitopes

Fourteen proteins were selected by the first criterion on the basis of belonging to a protein family of an established PAg. Seventy-two proteins were selected by the second criterion on the basis of having a biological process related to the virulence or latency of MTB. Upon merging the proteins selected by these criteria, 82 unique proteins remained.

The binding affinity of each of these 82 proteins to the reference sets of MHC-I and MHC-II alleles was predicted using IEDB binding prediction tools. The 82 selected proteins were ranked by the number of promiscuous epitopes for both MHC-I and MHC-II (Tables S3 and S4, respectively). To further refine our selection, we took the 11 proteins with the most promiscuous epitopes for both the MHC-I and MHC-II reference set of alleles, yielding 22 proteins. Because six of these proteins were found in both the MHC-I and MHC-II rankings, a total of 16 unique proteins remained in this final selection (Figure 1; Table 3).

## 4. Discussion

This study aimed to identify novel MTB PAgs to assist in the creation of a multistage TB vaccine strategy that will overcome the inadequacy of the BCG vaccine and confer broad immunity against both primary and post-primary TB in all populations. Upon filtering the H37Rv genome through Vaxign-ML, we selected 82 novel predicted PAgs that either belonged to protein families of previously established PAgs or were relevant to the virulence or latency of MTB. From this group, we then identified 16 predicted PAgs with the broadest immunogenic potential as indicated by the number of promiscuous MHC-I and MHC-II epitopes.

Although none of the identified 16 prospective MTB PAgs have previously been studied as vaccine candidates, several have been characterized to various degrees [17,50,51,52,53]. PE4 (Rv0160c) has previously been shown to exhibit elevated expression during cellular stress, including in the persistent stage of the MTB infection and has been described as an immunodominant antigen that elicits a strong humoral response in patients [50]. ClpB (Rv0384c) has been demonstrated to be required for the persistence of MTB bacilli within macrophages and under the stressful conditions of latency [51]. EccCa1 (Rv3870) has been shown to be essential for the secretion of ESAT-6 and CFP-10, proteins involved in MTB pathogenesis [52]. FadE5 (Rv0244c) was shown to be expressed to a similar degree in early- and late-stage TB and to be involved in the stress response [17]. Finally, PknD (Rv0931c) is believed to be essential for MTB infections of the central nervous system [53]. As none of these 16 proteins have been studied as vaccine candidates and many have not been previously characterized at all, they may be good candidates for future laboratory-based studies.

Of the 11 biological process categories that were identified as relevant to MTB virulence or LTBI, seven were represented among the 16 selected proteins, including interaction with the host immune system, cell envelope biogenesis and maintenance, growth in host, response to antibiotic, DNA repair, protein folding, and response to starvation (Table 3). Four of the 16 selected proteins, MmpL12 (Rv1522c), FadE5 (Rv0244c), FadD30 (Rv0404), and EccCa1 (Rv3870), are involved in interaction with the host immune system; these processes allow MTB to evade, modulate, or suppress a host’s immune system via mechanisms such as attenuation of macrophage antigen presentation to T-helper cells or downregulation of MHC-II gene expression, which can enable continued latency [8]. Three of the 16, PonA2 (Rv3682), FadD30 (Rv0404), and FadD15 (Rv2187), are involved in cell envelope biogenesis and maintenance; these processes are responsible for the unique cell envelope of MTB, which is critical to its slow intracellular growth, virulence, and innate impermeability to many drugs and antibiotics [28,29]. Three of the 16, Mce2D (Rv0592), EccCa1 (Rv3870), and Mce2A (Rv0589), are involved in growth in the host, a process self-evidently relevant to MTB’s virulence; many of the proteins involved in this process are considered virulence factors [32]. Three of the 16, IleS (Rv1536), RpoB (Rv0667), and PonA2 (Rv3682), are involved in the response to antibiotics; though proteins involved in antibiotic resistance are not themselves virulence factors, they can be instrumental in the persistence of disease [34]. One of the 16, UvrA (Rv1638), is involved in DNA repair, which is critical for the persistence of MTB in the hostile, oxidative environment of the macrophage [30]. One of the 16, ClpB (Rv0384c), is involved in protein folding; proteins that ensure correct protein folding, such as chaperonins, are also essential to MTB survival under stressful environments such as in the macrophage [33]. Finally, one of the 16, PknD (Rv0931c), is involved in response to starvation; because MTB faces both energy and nutrient starvation within the granuloma, proteins that enable persistence of the bacilli in spite of these low-nutrient conditions are also necessary for latency [37].

Among the 16 proteins selected on the basis of promiscuous epitopes, six are among those having the most promiscuous epitopes for both MHC-I and MHC-II alleles (Table 3). These six may provide the greatest immunogenic potential within this set of 16 proteins. However, it is worth noting that we exclusively consider the proteome of the H37Rv reference strain of MTB in this study. Taking into account the genetic diversity of MTB globally, it is important that proteins selected as vaccine candidates are highly conserved. Future studies of the genetic diversity of selected antigens using MTB clinical strains representing different genetic lineages are, therefore, warranted.

A major limitation of our study is that only the peptide sequence of each antigen was considered in our analyses. Our prediction did not consider higher-level protein structures or vaccine formulation, both of which may affect the interaction between the antigen and the host immune system, thereby impacting the induced immune response. A second major limitation is the absence of experimental validation of the protective antigenicity of the identified proteins with vaccine potential.

In light of these limitations, it is important to note that RV methods, such as Vaxign-ML, are not intended to replace laboratory-based immunological studies, but rather they are to serve in a complementary fashion. For example, in a lab setting it is not feasible to conduct a genome-wide search for protective antigens; RV methods, however, can quickly narrow the list of potential protective antigens, thereby informing the prioritization of targets for laboratory investigation. This narrowed list can then be tested and validated in a lab setting. Additionally, Vaxign-ML has previously been validated by demonstrating that all of the PAgs included in five recent MTB vaccines in clinical trials received Vaxign-ML protegenicity scores that met the threshold used in this study [23]. Similarly, it is worth noting that of the 25 proteins that have been established as MTB PAgs according to Protegen, 24 (recall = 0.96) of these received Vaxign-ML protegenicity scores that met the threshold for selection within this study.

To summarize, the bioinformatic approach applied in this study has allowed for the identification of 16 prospective novel MTB PAgs that may have been difficult to identify using traditional vaccinology techniques and that may be used in future subunit vaccines. For the selected antigens, all computational measures of immunogenicity and epitope promiscuity should, however, be further validated in vitro and in vivo. This integration of traditional and computational vaccine development tools may be the best approach in developing a broadly effective TB vaccine strategy that prevents both primary and post-primary TB in all populations and takes us closer to our ambition to End TB.

## Figures and Tables

**Table 1:** Novel Vaxign-ML-predicted PAgs that belong to protein families of established MTB PAgs.

Protein Family	Protein	Tuberculist ID
mycobacterial A85 antigen	FbpC (Ag85C)	Rv0129c
mycobacterial PE	PE_PGRS11	Rv0754
	PE4	Rv0160c
	PE26	Rv2519
mycobacterial PPE	Hypothetical protein Rv3822	Rv3822
	PPE28	Rv1800
	PPE8	Rv0355c
	PPE12	Rv0755c
	PPE30	Rv1802
peptidase S1C	HtrA	Rv1223
	PepD	Rv0983
PstS	PstS2	Rv0932c
RsiV	Hypothetical protein Rv3036c	Rv3036c
WXG100	EsxI	Rv1037c

**Table 2:** Novel Vaxign-ML-predicted PAgs having biological processes related to MTB virulence or latency. More than 72 proteins are listed here due to redundancy between categories.

Biological Process Category	Proteins (Tuberculist IDs)
Cell envelope biogenesis and maintenance	PonA2 (Rv3682), PonA1 (Rv0050), FadD15 (Rv2187), LdtB (Rv2518c), PbpB (Rv2163c), FadD30 (Rv0404), FbpC (Ag85C) (Rv0129c), AccD4 (Rv3799c), FadD32 (Rv3801c), hypothetical protein Rv3811 (Rv3811), LprQ (Rv0483), PbpA (Rv0016c), FadD19 (Rv3515c)
DNA Repair	RecA (Rv2737c), HtpG (Rv2299c), UvrA (Rv1638), LigD (Rv0938), RecG (Rv2973c), UvrB (Rv1633)
Interaction with host immune system	FadE5 (Rv0244c), Mce1A (Rv0169), probable aldehyde dehydrogenase (Rv0458), CaeA (Rv2224c), LprA (Rv1270c), FadD30 (Rv0404), EccCa1 (Rv3870), Icl1 (Rv0467), PknH (Rv1266c), MmpL12 (Rv1522c), UvrB (Rv1633), EccB1 (Rv3869), halimadienyl diphosphate synthase (Rv3377c), FadD19 (Rv3515c)
Fatty acid beta-oxidation	FadB (Rv0860), FadA3 (Rv1074c), Ltp1 (Rv2790c), probable nonspecific lipid-transfer protein (Rv1627c)
Growth in host	FadD13 (Rv3089), Mce2C (Rv0591), Mce4A (Rv3499c), Mce1A (Rv0169), Mce1F (Rv0174), Tgs4 (Rv3088), Mce3C (Rv1968), Mce1C (Rv0171), Mce3D (Rv1969), Mce3A (Rv1966), EccCa (Rv3870), Mce4C (Rv3497c), Mce2F (Rv0594), Mce4D (Rv3496c), Mce2A (Rv0589), EccA1 (Rv3868), Mce2D (Rv0592), Mce1D (Rv0172), FadA (Rv0243), Mce4F (Rv3494c)
Protein folding	GroEL2 (Rv0440), Mpa (Rv2115c), GroEL1 (Rv3417c), ClpX (Rv2457c), HtpG (Rv2299c), ClpB (Rv0384c)
Response to antibiotic	PonA2 (Rv3682), GyrB (Rv0005), RecA (Rv2737c), RpoB (Rv0667), PonA1 (Rv0050), FbpC (Ag85C) (Rv0129c), possible penicillin-binding lipoprotein (Rv2864c), PepD (Rv0983), IleS (Rv1536), LprG (Rv1411c)
Response to acidic pH	FadD13 (Rv3089), Tgs4 (Rv3088), Icl1 (Rv0467), AccD4 (Rv3799c)
Response to hypoxia	GroEL2 (Rv0440), PonA1 (Rv0050), Tgs4 (Rv3088), Icl1 (Rv0467), AccD4 (Rv3799c), PE_PGRS11 (Rv0754), Tuf (Rv0685), SdhA (Rv3318), probable succinate dehydrogenase (Rv0248c)
Response to nitrosative or oxidative stress	Mpa (Rv2115c), FtsH (Rv3610c), HtpG (Rv2299c), Tgs4 (Rv3088), Mpt53 (Rv2878c), AccD4 (Rv3799c), UvrB (Rv1633), CysN (Rv1286)
Response to starvation	halimadienyl diphosphate synthase (Rv3377c), PknD (Rv0931c), CysN (Rv1286)

**Table 3:** Final selection of prospective novel MTB vaccine candidates with sufficiently high Vaxign-ML protegenicity scores, belonging to the protein family of an established PAg and/or having a biological process related to the virulence or latency of MTB, and having the greatest number of MHC-I and/or MHC-II promiscuous epitopes.

Protein	Tuberculist ID	GO Biological Process	Subcellular Location	MHC-I Promiscuous Epitopes	MHC-II Promiscuous Epitopes
Having highest numbers of both MHC-I and MHC-II promiscuous epitopes					
PPE8	Rv0355c	not available	not available	104	194
IleS	Rv1536	isoleucyl-tRNA aminoacylation, response to antibiotics	cytoplasm	92	156
MmpL12	Rv1522c	response to host immune response	cell membrane, multi-pass membrane protein	86	263
UvrA	Rv1638	cellular response to DNA damage stimulus, negative regulation of strand invasion, nucleotide-excision repair, SOS response	cytoplasm	73	116
RpoB	Rv0667	response to antibiotic, DNA-templated transcription	cell wall, cytosol, plasma membrane	72	109
ClpB	Rv0384c	protein refolding, response to heat	cytoplasm	62	104
Having highest number of MHC-I promiscuous epitopes only					
PonA2	Rv3682	peptidoglycan biosynthetic process, response to antibiotic	not available	60	88
FadE5	Rv0244c	response to host immune response	extracellular region, plasma membrane	57	86
Mce2D	Rv0592	growth of symbiont in host, growth of symbiont in host vacuole	cell wall	57	84
FadD30	Rv0404	Actinobacterium-type cell wall biogenesis, fatty acid biosynthetic process, induction by symbiont of host immune response, lipid biosynthetic process	not available	56	85
EccCa1	Rv3870	evasion of host immune response, growth of symbiont in host, pathogenesis, protein secretion by the type VII secretion system	cell inner membrane, multi-pass membrane protein	56	79
Having highest number of MHC-II promiscuous epitopes only					
PPE28	Rv1800	not available	not available	54	176
PE4	Rv0160c	not available	not available	48	100
FadD15	Rv2187	Actinobacterium-type cell wall biogenesis, fatty acid biosynthetic process, lipid biosynthetic process, long-chain fatty acid metabolic process	cell wall, plasma membrane	48	113
PknD	Rv0931c	cellular response to phosphate starvation, negative regulation of catalytic activity, negative regulation of fatty acid biosynthetic process, negative regulation of protein binding, pathogenesis, positive regulation of catalytic activity	cell membrane, single-pass membrane protein	42	105
Mce2A	Rv0589	growth of symbiont in host, growth of symbiont in host vacuole	integral component of membrane	36	100

## Data Availability

All the data generated by the present study are provided either in the main body of the article or in its supplementary material.

## Supplementary Materials

The following are available online at https://www.mdpi.com/article/10.3390/vaccines9101098/s1, Table S1: Novel Vaxign-ML-predicted protective antigens, Table S2: Previously established MTB protective antigens as according to Protegen, Table S3: Proteins selected on the basis of belonging to the protein family of a previously established protective antigen or on the basis of having a GO biological process related to the virulence or latency of MTB, ranked by number of promiscuous MHC-I epitopes, Table S4: Proteins selected on the basis of belonging to the protein family of a previously established protective antigen or on the basis of having a GO biological process related to the virulence or latency of MTB ranked, by number of promiscuous MHC-II epitopes.
