# Supplementary material for: Identification of Mycobacterium tuberculosis Antigens with Vaccine Potential Using a Machine Learning-Based Reverse Vaccinology Approach
Source: Vaccines (Basel). 2021 Sep 28;9(10):1098. doi: 10.3390/vaccines9101098 (PMC8538456; doi:10.3390/vaccines9101098)
Supplement: Supplementary file 1 [file vaccines-09-01098-s001.zip › vaccines-1360802-supplementary.pdf]

Supplemental Table S1. Novel Vaxign-ML-predicted protective antigens.

Name	UniProt Accession Number	Tuberculist ID	Vaxign-ML Score	Molecular Function	GO Biological process	Subcellular Location	Protein Family
GroEL2	P9WPE7	Rv0440	98.88	chaperone	adhesion of symbiont to host, chaperone cofactor-dependent protein refolding, positive regulation of transcription regulatory region DNA binding, protein folding, protein refolding, response to heat, response to hypoxia	cytoplasm, secreted	chaperonin (HSP60) family
FusA1	P9WNM7	Rv0684	98.47	elongation factor in protein synthesis	ribosome disassembly	cytoplasm	classic translation factor GTPase family
PonA2	I6YGX2	Rv3682	98.47	carboxypetidase	peptidoglycan biosynthetic process, response to antibiotic	not available	not available
GyrB	P9WG45	Rv0005	98.24	topoisomerase	DNA-dependent DNA replication, DNA topological change, response to antibiotic	cytoplasm	type II topoisomerase GyrB family
SecA1	P9WGP5	Rv3240c	98.03	protein transport	intracellular protein transmembrane transport, protein import, protein targeting, protein transport by the Sec complex	cell membrane, cytoplasm	SecA family

Mpa	P9WQN5	Rv2115c	97.96	chaperone	cellular response to nitric oxide, modification-dependent protein catabolic process, pathogenesis, proteasomal protein catabolic process, proteasomal ubiquitin-independent protein catabolic process, protein unfolding, response to nitrosative stress	cell wall, plasma membrane	AAA ATPase family
GroEL1	P9WPE9	Rv3417c	97.73	chaperone	chaperone cofactor-dependent protein refolding, DNA protection, nucleoid organization, positive regulation of transcription regulatory region DNA binding, protein folding, protein refolding, response to heat	cytoplasm, cell surface	chaperonin (HSP60) family
PstS2	P9WGT9	Rv0932c	97.62	phosphate transport	pathogenesis, phosphate ion transmembrane transport, phosphate ion transport	cell membrane	PstS family
Acn	O53166	Rv1475c	97.55	lyase	citrate metabolic process, propionate metabolic process, methylcitrate cycle, response to iron ion, tricarboxylic acid cycle	cell wall, cytosol, plasma membrane	aconitase/IPM isomerase family
SahH	P9WGV3	Rv3248c	96.82	hydrolase	adenosine metabolic process, adhesion of symbiont to host cell, entry of bacterium into host cell, methionine catabolic process, one-carbon metabolic process, S-adenosylmethionine cycle	cytoplasm	adenosylhomocy steinase family

LpqK	P95207	Rv0399c	96.63	not available	not available	not available	not available
RpoC	P9WGY7	Rv0668	96.47	nucleotidyltransferase, transferase	transcription, DNA-templated	cell wall, plasma membrane	RNA polymerase beta' chain family
RecA	P9WHJ3	Rv2737c	96.20	DNA-binding, endonuclease, hydrolase, nuclease	cellular response to DNA damage stimulus, intein-mediated protein splicing, intron homing, recombinational repair, response to antibiotic, SOS response, strand invasion, UV protection	cytoplasm	RecA family
probable macrolide-transport ATP-binding protein ABC transporter	P9WQK3	Rv2477c	96.11	hydrolase, RNA-binding, rRNA-binding, tRNA-binding	negative regulation of translational elongation, translation	cytoplasm	ABCF family
ClpC1	P9WPC9	Rv3596c	96.11	chaperone	not available	cell wall, cytosol, plasma membrane	ClpA/ClpB family
FadD13	P9WQ37	Rv3089	95.92	ligase	fatty acid biosynthetic process, growth of symbiont in host cell, response to acidic pH	cell membrane	ATP-dependent AMP-binding enzyme family
EccA5	P9WPI1	Rv1798	95.88	ATPase	not available	cytoplasm	CbxX/CfxQ family
Mce2C	O07787	Rv0591	95.86	not available	growth of symbiont in host, growth of symbiont in host vacuole	cell wall	not available

HsdM	O33298	Rv2756c	95.86	methyltransferase, transferase	DNA methylation	cell wall, plasma membrane	N(4)/N(6)-methyltransferase family
FtsH	P9WQN3	Rv3610c	95.86	hydrolase, metalloprotease, protease	protein catabolic process, proteolysis, regulation of gene expression, response to oxidative stress	cell membrane, cytoplasmic side	AAA ATPase family, peptidase M41 family
Mce4A	I6YC99	Rv3499c	95.74	not available	growth of symbiont in host, growth of symbiont in host vacuole	cell wall	not available
RpoB	P9WGY9	Rv0667	95.72	nucleotidyltransferase, transferase	response to antibiotic, transcription, DNA-templated	cell wall, cytosol, plasma membrane	RNA polymerase beta chain family
FadE5	O53666	Rv0244c	95.69	oxidoreductase	response to host immune response	extracellular region, plasma membrane	acyl-CoA dehydrogenase family
Icd2	O53611	Rv0066c	95.65	oxidoreductase	glyoxylate cycle, isocitrate metabolic process, tricarboxylic acid cycle	cell wall, cytosol, extracellular region, plasma membrane	monomeric-type IDH family
Mce1A	Q79FZ9	Rv0169	95.33	actin binding	adhesion of symbiont to host, dormancy entry of symbiont in host, entry into host, evasion of host immune response via regulation of host cytokine network, growth of symbiont in host, growth of symbiont in host vacuole, heterophilic cell-cell adhesion via	cell wall, extracellular region, integral component of membrane, plasma membrane	not available

					plasma membrane cell adhesion molecules		
DppA	I6X811	Rv3666c	95.33	not available	peptide transport	not available	not available
ClpX	P9WPB9	Rv2457c	95.31	chaperone	cell division, negative regulation of protein polymerization, protein catabolic process, protein folding, proteolysis	cell wall	ClpX chaperone family
Mce1F	L0T2W6	Rv0174	95.24	not available	growth of symbiont in host, growth of symbiont in host vacuole	cell wall, extracellular region, integral component of membrane, plasma membrane	not available
TopA	P9WG49	Rv3646c	95.24	DNA-binding, isomerase, topoisomerase	DNA topological change, negative regulation of ribonuclease activity	cell wall, cytosol, plasma membrane	type IA topoisomerase family
LepA	P9WK97	Rv2404c	95.15	hydrolase	positive regulation of translation	cell membrane	classic translation factor GTPase family
SigA	P9WGI1	Rv2703	95.15	DNA-binding, sigma factor	pathogenesis, response to water, transcription initiation from bacterial-type RNA polymerase promoter	cytoplasm	sigma-70 factor family
PonA1	P71707	Rv0050	95.12	carboxypeptidase, glycosyltransferase, hydrolase, m	cellular response to hypoxia, cell wall organization, peptidoglycan	cell membrane	glycosyltransferase 51 family,

				ultifunctional enzyme, protease , transferase	biosynthetic process, regulation of cell shape, response to antibiotic		transpeptidase family
MetK	P9WGV1	Rv1392	95.12	transferase	one-carbon metabolic process, S-adenosylmethionine biosynthetic process	cytoplasm	AdoMet synthase family
FadD15	O53521	Rv2187	95.12	ligase	Actinobacterium-type cell wall biogenesis, fatty acid biosynthetic process, lipid biosynthetic process, long-chain fatty acid metabolic process	cell wall, plasma membrane	ATP-dependent AMP-binding enzyme family
GlnA1	P9WN39	Rv2220	95.12	ligase	glutamine biosynthetic process, nitrogen utilization, pathogenesis, positive regulation of plasminogen activation	cytoplasm	glutamine synthetase family
LdtB	I6Y9J2	Rv2518c	95.08	acyltransferase, tr ansferase	cell wall organization, peptidoglycan-protein cross-linking, regulation of cell shape	cell membrane	not available
FadE15	O53158	Rv1467c	94.99	oxidoreductase	not available	cell wall, plasma membrane	acyl-CoA dehydrogenase family
hypothetical protein Rv1461	P9WFP7	Rv1461	94.96	endonuclease, hy drolase, nuclease	intein-mediated protein splicing, intron homing, iron-sulfur cluster assembly	cytosol	UPF0051 (ycf24) family

hypothetical protein Rv1754c	O06790	Rv1754c	94.96	not available	not available	plasma membrane, transmembrane	not available
HtpG	P9WMJ7	Rv2299c	94.92	chaperone	cellular response to DNA damage stimulus, cellular response to superoxide, protein folding, response to heat	cytoplasm	heat shock protein 90 family
probable aldehyde dehydrogenase	P9WNY1	Rv0458	94.85	oxidoreductase	suppression by symbiont of host immune response	plasma membrane	aldehyde dehydrogenase family
MetH	O33259	Rv2124c	94.85	methyltransferase, transferase	methylation, pteridine-containing compound metabolic process	cell wall, cytosol, plasma membrane	vitamin-B12 dependent methionine synthase family
FprA	P9WIQ3	Rv3106	94.85	oxidoreductase	oxidation-reduction process	cell wall	ferredoxin--NADP reductase type 1 family
Tgs4	P9WKC3	Rv3088	94.78	acyltransferase, transferase	glycerolipid biosynthetic process, glycerol metabolic process, growth of symbiont in host cell, response to acidic pH, response to hypoxia, response to nitric oxide, triglyceride biosynthetic process	plasma membrane	long-chain O-acyltransferase family
ClpB	P9WPD1	Rv0384c	94.69	chaperone	protein refolding, response to heat	cytoplasm	ClpA/ClpB family

GlcB	P9WK17	Rv1837c	94.55	transferase	adhesion of symbiont to host, coenzyme A metabolic process, glyoxylate catabolic process, glyoxylate cycle, tricarboxylic acid cycle	cytoplasm	malate synthase family
PbpB	L0T911	Rv2163c	94.55	not available	cell wall organization, peptidoglycan biosynthetic process, regulation of cell shape	cell membrane	transpeptidase family
DlaT	P9WIS7	Rv2215	94.55	acyltransferase, a ntioxidant, transf erase	cell redox homeostasis, glycolytic process, pathogenesis	cell wall, cytosol, plasma membrane, pyruvate dehydrogenase complex	2-oxoacid dehydrogenase family
probable exported protein	P9WKQ1	Rv0888	94.53	hydrolase, porin	cellular response to lipid, hemolysis in other organism, ion transport, organic substance transport, sphingomyelin catabolic process	cell outer membrane	SpmT family
FadD25	P9WQ45	Rv1521	94.53	ligase	fatty acid biosynthetic process	cell membrane	ATP-dependent AMP-binding enzyme family
FadB	O53872	Rv0860	94.44	not available	fatty acid beta-oxidation	cell wall, cytosol, plasma membrane	not available
UvrA	P9WQK7	Rv1638	94.44	DNA-binding, Excision nuclease	cellular response to DNA damage stimulus, negative regulation of strand invasion, nucleotide-excision repair, SOS response	cytoplasm	UvrA family

CaeA	P9WHR3	Rv2224c	94.44	hydrolase	pathogenesis, post-translational protein modification, proteolysis, response to host immune response	cell membrane	peptidase S33 family
LpqL	P96264	Rv0418	94.11	aminopeptidase, hydrolase, protea se	proteolysis	cell membrane	peptidase M28 family
Mce3C	O53969	Rv1968	94.11	not available	growth of symbiont in host, growth of symbiont in host vacuole	cell wall	not available
FadE19	I6Y0W5	Rv2500c	94.11	oxidoreductase	not available	plasma membrane	acyl-CoA dehydrogenase family
EccC2	O05450	Rv3894c	94.11	not available	not available	cell wall, plasma membrane	not available
FadA3	O53422	Rv1074c	94.05	acyltransferase, transferase	fatty acid beta-oxidation	cell wall, extracellular region, plasma membrane	Thiolase family
InfB	P9WKK1	Rv2839c	94.05	initiation factor	translational initiation	cytoplasm	classic translation factor GTPase family
Mce1C	O07415	Rv0171	93.84	not available	growth of symbiont in host, growth of symbiont in host vacuole	cell wall, extracellular region	not available

TypA	O06563	Rv1165	93.75	elongation factor	protein biosynthesis	cell wall, plasma membrane, ribonucleoprotein complex	classic translation factor GTPase family
Probable conserved transmembrane protein	P9WFL3	Rv3193c	93.63	not available	not available	cell membrane, multi-pass membrane protein	UPF0182 family
Multifunctional alpha-ketoglutarate metabolic enzyme	P9WIS5	Rv1248c	93.61	acyltransferase, allosteric enzyme, decarboxylase, lyase, multifunctional enzyme, oxidoreductase, transferase	2-oxoglutarate metabolic process, oxidation-reduction process, tricarboxylic acid cycle	cell wall, cytosol, oxoglutarate dehydrogenase complex, plasma membrane, pyruvate dehydrogenase complex	2-oxoacid dehydrogenase family
LprA	P9WK55	Rv1270c	93.59	not available	evasion of host immune response via regulation of host cytokine network	cell membrane	LppX/LprAFG lipoprotein family
SirA	P9WJ03	Rv2391	93.59	oxidoreductase	cysteine biosynthetic process from serine, sulfate assimilation	cell wall, cytosol	nitrite and sulfite reductase 4Fe-4S domain family
ChoD	P9WMV9	Rv3409c	93.43	isomerase, oxidoreductase	cholesterol metabolic process, pathogenesis, steroid biosynthetic process	not available	GMC oxidoreductase family
AtsD	I6XVW9	Rv0663	93.36	hydrolase	not available	not available	sulfatase family

hypothetical protein Rv2721c	I6XF52	Rv2721c	93.29	not available	not available	integral component of membrane	not available
Mpt53	P9WG65	Rv2878c	93.27	oxidoreductase	cell redox homeostasis, cellular response to oxidative stress, oxidation-reduction process	secreted	thioredoxin family
Mce3D	O53970	Rv1969	93.18	not available	growth of symbiont in host, growth of symbiont in host vacuole	cell wall	not available
LppF	O53963	Rv1921c	93.13	not available	not available	not available	not available
FadD30	P9WQ57	Rv0404	93.11	ligase	Actinobacterium-type cell wall biogenesis, fatty acid biosynthetic process, induction by symbiont of host immune response, lipid biosynthetic process	not available	ATP-dependent AMP-binding enzyme family
Mce3A	L7N698	Rv1966	93.11	not available	growth of symbiont in host, growth of symbiont in host vacuole	integral component of membrane	not available
PlsC	I6YDI9	Rv2483c	93.11	acyltransferase	phosphatidic acid biosynthetic process	plasma membrane	SerB family
Icd1	P9WKL1	Rv3339c	93.11	oxidoreductase	glyoxylate cycle, isocitrate metabolic process, NADP metabolic process, tricarboxylic acid cycle	plasma membrane	isocitrate and isopropylmalate dehydrogenases family
EccCa1	P9WNB3	Rv3870	93.02	not available	evasion of host immune response, growth of symbiont in host,	cell inner membrane, multi-	not available

					pathogenesis, protein secretion by the type VII secretion system	pass membrane protein	
SthA	P9WHH5	Rv2713	92.97	oxidoreductase	cell redox homeostasis, NADP metabolic process, oxidation-reduction process	cytoplasm	class-I pyridine nucleotide-disulfide oxidoreductase family
probable aldehyde dehydrogenase	I6X8S7	Rv0223c	92.88	oxidoreductase	not available	not available	aldehyde dehydrogenase family
DnaA	P9WNW3	Rv0001	92.83	DNA-binding	ADP biosynthetic process, dephosphorylation, DNA replication, DNA replication initiation, regulation of DNA replication	cytoplasm	DnaA family
Icl1	P9WKK7	Rv0467	92.83	lyase	cellular response to hypoxia, glyoxylate cycle, isocitrate metabolic process, pathogenesis, response to acetate, response to acidic pH, response to fatty acid, response to host immune response, symbiont tolerance to host environment, tricarboxylic acid cycle	cytosol, extracellular region, plasma membrane	Isocitrate lyase family
CpsY	P9WGD1	Rv0806c	92.83	transferase	polysaccharide biosynthetic process	cell wall	stealth family

GltA2	P9WPD5	Rv0896	92.81	allosteric enzyme, transfera se	tricarboxylic acid cycle	cytosol, plasma membrane	citrate synthase family
IlvB1	P9WG41	Rv3003c	92.81	transferase	branched-chain amino acid biosynthetic process, isoleucine biosynthetic process, valine biosynthetic process	acetolactate synthase complex, cell wall	TPP enzyme family
GabD1	P9WNX9	Rv0234c	92.76	oxidoreductase	tricarboxylic acid cycle	plasma membrane	aldehyde dehydrogenase family
NirB	O53674	Rv0252	92.76	oxidoreductase	nitrate assimilation	cell wall, cytosol, plasma membrane	not available
FadD2	P95227	Rv0270	92.70	ligase	not available	cell wall, plasma membrane	not available
AtpA	P9WPU7	Rv1308	92.67	translocase	ATP synthesis coupled proton transport	cell wall, plasma membrane, proton-transporting ATP synthase complex, catalytic core F(1)	ATPase alpha/beta chains family
Pgk	P9WID1	Rv1437	92.67	kinase, transferas e	gluconeogenesis, glycolytic process, positive regulation of oxidative phosphorylation	cytoplasm	phosphoglycerate kinase family
LigD	P9WNV3	Rv0938	92.63	DNA-binding, DNA-directed DNA	DNA ligation, DNA recombination, DNA replication, synthesis of RNA primer, double-strand break repair,	plasma membrane	LigD polymerase family, LigD 3'-phosphoesterase

				polymerase, exonuclease, hydrolase, ligase, multifunctional enzyme, nuclease, nucleotidyltransferase	double-strand break repair via nonhomologous end joining, viral process		family, ATP-dependent DNA ligase
PknH	P9WI71	Rv1266c	92.63	kinase, serine/threonine-protein kinase, transferase	negative regulation of catalytic activity, negative regulation of growth, positive regulation of catalytic activity, positive regulation of DNA binding, positive regulation of transcription, DNA-templated, protein autophosphorylation, regulation of lipid biosynthetic process, response to host immune response	cell membrane, single-pass membrane protein	Ser/Thr protein kinase family
NrdE	P9WH75	Rv3051c	92.63	allosteric enzyme, oxidoreductase	deoxyribonucleotide biosynthetic process, DNA replication, oxidation-reduction process	plasma membrane, ribonucleoside-diphosphate reductase complex	ribonucleoside diphosphate reductase large chain family
HtrA	O06291	Rv1223	92.58	hydrolase, protease	not available	transmembrane	peptidase S1C family
FbpC (Ag85C)	P9WQN9	Rv0129c	92.53	acyltransferase, transferase	glycolipid biosynthetic process, lipid transport, mycolate cell wall layer assembly, response to antibiotic	secreted	mycobacterial A85 antigen family

probable iron-sulfur-binding reductase	O33268	Rv0338c	92.51	oxidoreductase	not available	transmembrane	not available
AccD4	O53578	Rv3799c	92.51	ligase	carbon fixation, mycolate cell wall layer assembly	cell wall, plasma membrane	AccD/PCCB family
NarH	O06560	Rv1162	92.47	not available	anaerobic respiration, cellular response to decreased oxygen levels, cellular response to nitrate, cellular response to nitric oxide, nitrate metabolic process, response to acidic pH	cell wall, plasma membrane	not available
TB22.2	I6YF08	Rv3036c	92.47	hydrolase	lipid metabolic process	cell membrane, single-pass membrane protein, cell wall	RsiV family
MycP5	O53945	Rv1796	92.40	hydrolase, protea se, serine protease	protein processing	cell membrane, single-pass membrane protein	peptidase S8 family
probable PhiRv1 integrase	O06604	Rv1586c	92.37	not available	DNA recombination	cell wall	not available
MycP2	O05458	Rv3886c	92.37	hydrolase, protea se, serine protease	protein processing	cell membrane, single-pass membrane protein	peptidase S8 family

PlcB	P9WIB3	Rv2350c	92.31	hydrolasae	disruption by symbiont of host cellular component, pathogenesis	cell membrane; peripheral membrane protein; extracellul ar side	bacterial phospholipase C family
LppH	I6YGJ4	Rv3576	92.31	not available	not available	cell membrane	not available
hypothetical protein Rv3822	O07801	Rv3822	92.24	acyltransferase, tr ansferase	lipid metabolic process	cell membrane, single-pass membrane protein	mycobacterial PPE family
possible transposase	O07796	Rv3827c	92.21	not available	not available	plasma membrane	not available
possible penicillin-binding lipoprotein	O33346	Rv2864c	92.17	not available	cell wall organization, response to antibiotic	cell wall, integral component of plasma membrane	not available
MmpL12	P9WJT7	Rv1522c	92.12	not available	response to host immune response	cell membrane, multi-pass membrane protein	resistance-nodulation-cell division (RND) (TC 2.A.6) family
PlcA	P9WIB5	Rv2351c	92.12	hydrolase	disruption by symbiont of host cellular component, pathogenesis	cell membrane; periphe ral membrane protein; extracellul ar side	bacterial phospholipase C family

FadD32	O53580	Rv3801c	92.12	ligase	Actinobacterium-type cell wall biogenesis, fatty acid biosynthetic process, lipid biosynthetic process, mycolate cell wall layer assembly	cell wall, plasma membrane	ATP-dependent AMP-binding enzyme family
RplA	P9WHC7	Rv0641	92.10	repressor, ribonucleoprotein, ribosomal protein, RNA-binding, rRNA-binding, tRNA-binding	maturation of LSU-rRNA, regulation of translation, translation	cell wall, cytosol, cytosolic large ribosomal subunit, plasma membrane	universal ribosomal protein uL1 family
PE_PGRS11	Q79FW5	Rv0754	92.10	isomerase	glycolytic process, pathogenesis, response to hypoxia	cell wall, cell surface	mycobacterial PE family, phosphoglycerate mutase family
FadE21	I6XFA9	Rv2789c	92.10	oxidoreductase	not available	plasma membrane	acyl-CoA dehydrogenase family
conserved protein Rv2823c	P71629	Rv2823c	92.10	endonuclease, exonuclease, hydrolase, RNA-binding, transferase	defense response to virus	extracellular region, plasma membrane	CRISPR-associated Cas10/Csm1 family
Ltp1	O33332	Rv2790c	92.08	acyltransferase, transferase	fatty acid beta-oxidation	cell wall	thiolase family

MetS	P9WFU5	Rv1007c	92.03	aminoacyl-tRNA synthetase, ligase	methionyl-tRNA aminoacylation	cytoplasm	class-I aminoacyl-tRNA synthetase family
Cfp2 (MTB12)	P9WIN7	Rv2376c	92.01	not available	not available	secreted	MTB12 family
AccD5	P9WQH7	Rv3280	91.99	ligase	carbon fixation	acetyl-CoA carboxylase complex, cell wall, plasma membrane, protein-containing complex	AccD/PCCB family
ValS	P9WFS9	Rv2448c	91.89	aminoacyl-tRNA synthetase, ligase	tRNA aminoacylation for protein translation, valyl-tRNA aminoacylation	cytoplasm	class-I aminoacyl-tRNA synthetase family
Mce4C	I6YGB1	Rv3497c	91.89	not available	growth of symbiont in host, growth of symbiont in host vacuole	cell wall	not available
PepD	O53896	Rv0983	91.87	hydrolase, protease	cellular response to antibiotic, pathogenesis, protein catabolic process, proteolysis	integral component of membrane	peptidase S1C family
FadD11	P9WQ53	Rv1550	91.87	ligase	fatty acid metabolic process	cell membrane, multi-pass membrane protein	ATP-dependent AMP-binding enzyme family
AtpD	P9WPU5	Rv1310	91.83	translocase	ATP synthesis coupled proton transport	cell membrane, peripheral membrane protein	ATPase alpha/beta chains family

RpsA	P9WH43	Rv1630	91.83	ribonucleoprotein, ribosomal protein, RNA-binding	translation	cell wall, cytosol, cytosolic small ribosomal subunit, plasma membrane	bacterial ribosomal protein bS1 family
GuaB2	P9WKI7	Rv3411c	91.83	oxidoreductase	GMP biosynthetic process, GTP biosynthetic process, IMP catabolic process, XMP biosynthetic process	cell wall, plasma membrane	IMPDH/GMPR family
CpsA	O06347	Rv3484	91.83	not available	not available	integral component of membrane	LytR/CpsA/Psr (LCP) family
hypothetical protein Rv3811	Q79F96	Rv3811	91.83	not available	peptidoglycan catabolic process	not available	N-acetylmuramoyl-L-alanine amidase 2 family
Gap	P9WN83	Rv1436	91.78	oxidoreductase	glucose metabolic process, glycolytic process	cytolplasm	glyceraldehyde-3-phosphate dehydrogenase family
ThrS	P9WFT5	Rv2614c	91.76	aminoacyl-tRNA synthetase, ligase, RNA-binding, tRNA-binding	threonyl-tRNA aminoacylation	cytolplasm	class-II aminoacyl-tRNA synthetase family
FusA2	P9WNM9	Rv0120c	91.73	not available	ribosome disassembly	cell wall, plasma membrane	classic translation factor GTPase family

probable aldehyde dehydrogenase	P96824	Rv0147	91.71	oxidoreductase	cellular aldehyde metabolic process	cell wall, plasma membrane	aldehyde dehydrogenase family
PurA	P9WHN3	Rv0357c	91.71	ligase	'de novo' AMP biosynthetic process, IMP metabolic process	cytoplasm	adenylosuccinate synthetase family
LprQ	P9WKV3	Rv0483	91.71	acyltransferase, transferase	cell wall organization, peptidoglycan-protein cross-linking, regulation of cell shape	extracellular region	not available
possible oxygenase	P95277	Rv1937	91.71			cell wall, cytosol, plasma membrane	not available
QcrA	P9WH23	Rv2195	91.71	oxidoreductase	not available	cell membrane, multi-pass membrane protein	Rieske iron-sulfur protein family
Ffh	P9WGD7	Rv2916c	91.69	ribonucleoprotein, RNA-binding	SRP-dependent cotranslational protein targeting to membrane	cytoplasm	GTP-binding SRP family
Mce2F	O07784	Rv0594	91.66	not available	growth of symbiont in host, growth of symbiont in host vacuole	integral component of membrane	not available
LpqW	P9WGU7	Rv1166	91.62	not available	glycolipid biosynthetic process, pathogenesis, peptide transport, phosphatidylinositol metabolic process, phospholipid biosynthetic process	extracellular region, outer membrane-bounded periplasmic space	bacterial solute-binding protein 5 family

hypothetical protein Rv2744c	P9WHP5	Rv2744c	91.62	not available	not available	cytoplasm	PspA/IM30 family
FadE24	P95187	Rv3139	91.62	oxidoreductase	not available	cell wall, plasma membrane	acyl-CoA dehydrogenase family
IleS	P9WFV3	Rv1536	91.55	aminoacyl-tRNA synthetase, ligase	isoleucyl-tRNA aminoacylation, response to antibiotics	cytoplasm	class-I aminoacyl-tRNA synthetase family
Tuf	P9WNN1	Rv0685	91.53	elongation factor	response to hypoxia, response to iron ion, translational elongation	cytoplasm	classic translation factor GTPase family
RipA	O53168	Rv1477	91.53	hydrolase, protea se, thiol protease	cell wall organization or biogenesis	secreted	peptidase C40 family
EccC5	P9WNA5	Rv1783	91.53	not available	not available	cell inner membrane, multi-pass membrane protein	not available
Mqo	P9WJP5	Rv2852c	91.53	oxidoreductase	tricarboxylic acid cycle	cell wall, plasma membrane	MQO family
probable transposase	I6Y263	Rv2978c	91.53	DNA-binding	DNA recombination, transposition	not available	transposase 35 family, transposase 2 family

probable ABC transporter ATP-binding protein	O53343	Rv3197	91.53	not available	not available	cell wall	not available
PE4	L7N661	Rv0160c	91.44	not available	not available	not available	
EsxI	P0DOA6	Rv1037c	91.44	not available	not available	secreted	WXG100 family
hypothetical protein Rv2190c	P9WHU3	Rv2190c	91.41	hydrolase, protease, thiol protease	not available	extracellular region	peptidase C40 family
Mce4D	I6XHD6	Rv3496c	91.41	not available	growth of symbiont in host, growth of symbiont in host vacuole	cell wall	not available
DnaE1	P9WNT7	Rv1547	91.39	DNA-directed DNA polymerase, nucleotidyltransferase, transferase	DNA replication	cytoplasm	DNA polymerase type-C family
probable transposase	O33333	Rv2791c	91.39	DNA-binding	DNA recombination, transposition	cell wall, plasma membrane	transposase 35 family, transposase 2 family
hypothetical protein Rv2787	O33329	Rv2787	91.37	not available	negative regulation of cell division	cell wall, cytoplasmic side of plasma membrane, cytosol, plasma membrane	not available

FadD8	O06417	Rv0551c	91.34	ligase	not available	plasma membrane	ATP-dependent AMP-binding enzyme family
FadE23	P95186	Rv3140	91.34	oxidoreductase	not available	cell wall, plasma membrane	acyl-CoA dehydrogenase family
PPE28	P9WI11	Rv1800	91.28	not available	not available	not available	mycobacterial PPE family
RecG	P9WMQ7	Rv2973c	91.28	DNA-binding, helicase, hydrolase	DNA recombination, DNA repair	plasma membrane	helicase family
PbpA	P9WKD1	Rv0016c	91.16	not available	cell wall organization, peptidoglycan biosynthetic process, regulation of cell shape	cell membrane, Single-pass type II membrane protein	transpeptidase family
probable carbon monoxide dehydrogenase large subunit	O53708	Rv0373c	91.16	carbon-monoxide dehydrogenase (acceptor) activity	not available	plasma membrane	not available
possible conserved exported protein	O86365	Rv0584	91.16	glycosidase, hydrolase	glycoprotein catabolic process, protein deglycosylation, protein quality control for misfolded or incompletely synthesized proteins	cytosol	glycosyl hydrolase 92 family
GpsI	P9WI57	Rv2783c	91.16	nucleotidyltransferase, RNA-	mRNA catabolic process, RNA catabolic process, RNA processing	cytoplasm	polyribonucleotide

				binding, transferase			nucleotidyltransferase family
hypothetical protein Rv1945	P9WLQ5	Rv1945	91.14	not available	not available	not available	Rv1128c/1148c/1588c/1702c/1945/3466 family
possible transposase	I6Y560	Rv0922	91.12	not available	not available	not available	not available
Rho	P9WHF3	Rv1297	91.12	helicase, hydrolase, RNA-binding	termination of RNA polymerase I transcription	cell wall, plasma membrane	Rho family
ProX	O69725	Rv3759c	91.12	not available	glycine betaine transport, response to host	ATP-binding cassette (ABC) transporter complex, extracellular region	not available
FabG4	I6Y778	Rv0242c	91.09	oxidoreductase	oxidation-reduction process	not available	short-chain dehydrogenases/ reductases (SDR) family
PlcC	P9WIB1	Rv2349c	91.09	hydrolase	disruption by symbiont of host cellular component, pathogenesis	not available	bacterial phospholipase C family
hypothetical protein Rv1006	O05592	Rv1006	91.07	not available	not available	cell wall, extracellular region, plasma membrane	not available

Mce2A	Q79FY7	Rv0589	91.05	not available	growth of symbiont in host, growth of symbiont in host vacuole	integral component of membrane	not available
probable transposase	I6Y941	Rv0920c	91.00	DNA-binding	transposition, DNA-mediated	not available	transposase mutator family
Cbs	P9WP51	Rv1077	91.00	lyase	cysteine biosynthetic process from serine, cysteine biosynthetic process via cystathionine	cell wall, cytoplasm, extracellular region, plasma membrane	cysteine synthase/cystathionine beta-synthase family
UvrB	P9WFC7	Rv1633	91.00	excision nuclease	induction by symbiont of host immune response, nucleotide-excision repair, response to nitrosative stress, SOS response	cytoplasm	UvrB family
hypothetical protein Rv2164c	O06213	Rv2164c	91.00	not available	not available	integral component of membrane	not available
PepB	P9WHT3	Rv2213	91.00	aminopeptidase, hydrolase, protease	not available	cytoplasm	peptidase M17 family
PE26	Q79FD3	Rv2519	91.00	aspartic-type endopeptidase activity	not available	not available	not available
UgpB	P71619	Rv2833c	91.00	not available	not available	plasma membrane	not available

hypothetical protein Rv3267	P96872	Rv3267	91.00	not available	not available	extracellular region, plasma membrane	LytR/CpsA/Psr (LCP) family
SdhA	O53370	Rv3318	91.00	oxidoreductase	anaerobic respiration, tricarboxylic acid cycle	cell inner membrane, peripheral membrane protein, cytoplasmic side	FAD-dependent oxidoreductase 2 family
EccA1	P9WPH9	Rv3868	91.00	not available	growth of symbiont in host	cytoplasm	CbxX/CfxQ family
EccB1	P9WNR7	Rv3869	91.00	hydrolase	modulation by symbiont of host immune response	cell inner membrane, single-pass membrane protein	EccB family
probable aminotransferase	O53379	Rv3329	90.98	aminotransferase, transferase	biotin biosynthetic process	not available	class-III pyridoxal-phosphate-dependent aminotransferase family
Mce2D	I6WYT7	Rv0592	90.95	not available	growth of symbiont in host, growth of symbiont in host vacuole	cell wall	not available
RpfA	P9WG31	Rv0867c	90.95	hydrolase	negative regulation of gene expression, pathogenesis, positive regulation of growth rate	extracellular region	transglycosylase family

AccD1	I6YDK7	Rv2502c	90.95	ligase	leucine catabolic process	methylcrotonoyl-CoA carboxylase complex	AccD/PCCB family
PpiB	P9WHW1	Rv2582	90.95	isomerase, rotam ase	not available	integral component of plasma membrane	cyclophilin-type PPIase family
halimadienyl diphosphate synthase	O50406	Rv3377c	90.95	isomerase, lyase	cellular response to magnesium starvation, geranylgeranyl diphosphate metabolic process, response to host immune response, tuberculosinol biosynthetic process	not available	terpene synthase family
Mce1D	O07416	Rv0172	90.93	not available	growth of symbiont in host, growth of symbiont in host vacuole	integral component of membrane	not available
FadA2	O86361	Rv0243	90.93	acyltransferase, transferase	growth of symbiont in host cell	cell wall, cytosol, plasma membrane	Thiolase family
probable succinate dehydrogenase	O53670	Rv0248c	90.93	oxidoreductase	anaerobic respiration	cell wall, plasma membrane	not available
PPE8	I6Y7L4	Rv0355c	90.93	not available	not available	not available	mycobacterial PPE family
PPE12	P9WI37	Rv0755c	90.93	not available	not available	not available	mycobacterial PPE family

hypothetical protein Rv0822c	I6WZI4	Rv0822c	90.93	not available	not available	not available	LytR/CpsA/Psr (LCP) family
PknD	P9WI79	Rv0931c	90.93	kinase, serine/threonine-protein kinase, transferase	cellular response to phosphate starvation, negative regulation of catalytic activity, negative regulation of fatty acid biosynthetic process, negative regulation of protein binding, pathogenesis, positive regulation of catalytic activity	cell membrane, single-pass membrane protein	Ser/Thr protein kinase family
CysN	P9WNM5	Rv1286	90.93	kinase, multifunctional enzyme, nucleotidyltransferase, transferase	cellular response to oxidative stress, cellular response to sulfur starvation, hydrogen sulfide biosynthetic process, sulfate assimilation, sulfate assimilation via adenylyl sulfate reduction	cytosol, plasma membrane, sulfate adenylyltransferase complex (ATP)	APS kinase family, classic translation factor GTPase family
CarB	P9WPK3	Rv1384	90.93	ligase	'de novo' UMP biosynthetic process, arginine biosynthetic process, glutamine metabolic process, nitrogen compound metabolic process	cytoplasm, plasma membrane	CarB family
LprG	P9WK45	Rv1411c	90.93	glycolipid binding, phosphatidylinositol binding	lipid transport, pathogenesis, response to antibiotic	cell inner membrane; lipid-anchor, cell wall, secreted, cell surface	LppX/LprAFG lipoprotein family

Zwf2	P9WN73	Rv1447c	90.93	oxidoreductase	glucose metabolic process, pentose-phosphate shunt	plasma membrane	glucose-6-phosphate dehydrogenase family
PykA	P9WKE5	Rv1617	90.93	kinase, transferase	glycolytic process	cytoplasm, cytosol, plasma membrane	pyruvate kinase family
probable nonspecific lipid-transfer protein	O06144	Rv1627c	90.93	acetyl-CoA C-acetyltransferase activity	fatty acid beta-oxidation	cell wall, plasma membrane	not available
PPE30	P9WI07	Rv1802	90.93	not available	not available	not available	mycobacterial PPE family
hypothetical protein Rv2264c	O53538	Rv2264c	90.93	not available	not available	extracellular region	not available
GgtB	P71750	Rv2394	90.93	transferase	not available	extracellular region, plasma membrane	not available
AlaS	P9WFW7	Rv2555c	90.93	aminoacyl-tRNA synthetase, ligase, RNA-binding, tRNA-binding	alanyl-tRNA aminoacylation, tRNA modification	cytoplasm	class-II aminoacyl-tRNA synthetase family

FtsK	P9WNA3	Rv2748c	90.93	DNA-binding	cell cycle, cell division, chromosome segregation	cell membrane, multi-pass membrane protein	FtsK/SpoIIIE/SftA family
ProS	P9WFT9	Rv2845c	90.93	aminoacyl-tRNA synthetase, ligase	prolyl-tRNA aminoacylation	cytoplasm	class-II aminoacyl-tRNA synthetase family
probable transposase	P9WL37	Rv2885c	90.93	DNA-binding	DNA recombination, transposition	plasma membrane	transposase 2 family, transposase 35 family
GatA	P9WQA1	Rv3011c	90.93	ligase	translation	glutamyl-tRNA(Gln) amidotransferase complex	amidase family
Cyp136	P9WPM7	Rv3059	90.93	monooxygenase, oxidoreductase	oxidation-reduction process, sterol metabolic process	not available	cytochrome P450 family
hypothetical protein Rv3194c	O53340	Rv3194c	90.93	hydrolase, protease, serine protease	protein catabolic process	extracellular region	peptidase S16 family
Mce4F	I6YC95	Rv3494c	90.93	not available	growth of symbiont in host, growth of symbiont in host vacuole	cell wall	not available
FadD19	P9WQ51	Rv3515c	90.93	ligase	Actinobacterium-type cell wall biogenesis, cholesterol metabolic process, fatty acid biosynthetic	cell wall	ATP-dependent AMP-binding enzyme family

					process, lipid biosynthetic process, response to host immune response		
Ltp3	I6YGD8	Rv3523	90.93	transferase activity, transferring acyl groups other than amino-acyl groups	not available	not available	not available
Pks13	I6X8D2	Rv3800c	90.93	multifunctional enzyme, transfera se	biosynthetic process	not available	not available
EthA	P9WNF9	Rv3854c	90.93	monooxygenase, oxidoreductase	drug metabolic process, oxidation-reduction process	cell membrane	FAD-binding monooxygenase family
EspI	P9WJC5	Rv3876	90.93	ATPase activity	negative regulation of cell division, pathogenesis, protein secretion by the type VII secretion system	cytoplasmic side of plasma membrane, cytosol, plasma membrane	not available

Supplemental Table S2. Previously established MTB protective antigens as according to Protegen.

Name	UniProt Accession Number	Tuberculist ID	Vaxign-ML Score	Protein Family
DnaK	P9WMJ9	Rv0350	98.42	heat shock protein 70 family
Mpt64	P9WIN9	Rv1980c	97.07	RsiV family
PstS3	P9WGT7	Rv0928	97.05	PstS family
KatG	I6YBX7	Rv1908c	96.13	peroxidase family
PstS1	P9WGU1	Rv0934	95.86	PstS family
Mpt63	P9WIP1	Rv1926c	95.42	not available
FbpB (Ag85B)	P9WQP1	Rv1886c	95.21	mycobacterial A85 antigen family
FbpD (Ag85C)	P9WQN7	Rv3803c	95.12	mycobacterial A85 antigen family
EsxA	P9WNK7	Rv3875	94.89	WXG100 family
EsxB	P9WNK5	Rv3874	94.30	WXG100 family
PepA	O07175	Rv0125	94.28	peptidase S1C family
Mpt83	P9WNF3	Rv2873	94.25	not available

Apa (Mpt32)	P9WIR7	Rv1860	94.21	Apa family
HbhA	P9WIP9	Rv0475	93.93	not available
FbpA (Ag85A)	P9WQP3	Rv3804c	92.37	mycobacterial A85 antigen family
BfrA	P9WPQ9	Rv1876	92.08	bacterioferritin family
PPE18	L7N675	Rv1196	92.05	mycobacterial PPE family
PPE42	P9WHZ5	Rv2608	91.89	mycobacterial PPE family
PE20	L7N656	Rv1806	91.64	mycobacterial PE family
EsxV	P0DOA7	Rv3619c	91.41	WXG100 family
hypothetical protein Rv2660c	I6Y1F5	Rv2660c	91.23	not available
hypothetical protein Rv1813c	P9WLS1	Rv1813c	91.09	not available
EsxW	P9WNI3	Rv3620c	90.95	WXG100 family
PPE14	P9WI33	Rv0915c	90.93	mycobacterial PPE family
PPE31	L0T7Y7	Rv1807	not selected by Vaxign-ML	mycobacterial PPE family

Supplemental Table S3. Proteins selected on the basis of belonging to the protein family of a previously established protective antigen or on the basis of having a GO biological process related to the virulence or latency of MTB, ranked by number of promiscuous MHC-I epitopes.

Name	Tuberculist ID	MHC-I Promiscuous Epitopes
PPE8	Rv0355c	104
IleS	Rv1536	92
MmpL12	Rv1522c	86
UvrA	Rv1638	73
RpoB	Rv0667	72
ClpB	Rv0384c	62
PonA2	Rv3682	60
FadE5	Rv0244c	57
Mce2D	Rv0592	57
FadD30	Rv0404	56
EccCa1	Rv3870	56
LigD	Rv0938	55
RecG	Rv2973c	55

PPE28	Rv1800	54
UvrB	Rv1633	53
PonA1	Rv0050	52
HtpG	Rv2299c	51
EccA1	Rv3868	50
PE4	Rv0160c	48
PbpB	Rv2163c	48
FadD15	Rv2187	48
FtsH	Rv3610c	48
PE_PGRS11	Rv0754	46
possible penicillin-binding lipoprotein	Rv2864c	45
FadD32	Rv3801c	45
GyrB	Rv0005	44
Mce1D	Rv0172	44
RecA	Rv2737c	44

FadD13	Rv3089	44
Mce4F	Rv3494c	44
AccD4	Rv3799c	44
Mce3A	Rv1966	43
Tgs4	Rv3088	43
EccB1	Rv3869	43
probable succinate dehydrogenase	Rv0248c	42
Mce2C	Rv0591	42
PknD	Rv0931c	42
Mce3D	Rv1969	42
Mce1A	Rv0169	41
Mce1C	Rv0171	41
CysN/	Rv1286	41
probable aldehyde dehydrogenase	Rv0458	40
Mce2F	Rv0594	40

HtrA	Rv1223	40
Mce3C	Rv1968	40
halimadienyl diphosphate synthase	Rv3377c	39
PbpA	Rv0016c	38
FadB	Rv0860	38
Mpa	Rv2115c	38
ClpX	Rv2457c	37
Mce1F	Rv0174	36
LprQ	Rv0483	36
Mce2A	Rv0589	36
CaeA	Rv2224c	36
FadD19	Rv3515c	36
FadA2	Rv0243	34
Icl1	Rv0467	34
Mce4D	Rv3496c	34

PPE30	Rv1802	33
hypothetical protein Rv3811	Rv3811	33
PE26	Rv2519	32
Mce4C	Rv3497c	32
Mce4A	Rv3499c	32
FbpC (Ag85C)	Rv0129c	31
GroEL2	Rv0440	30
SdhA	Rv3318	30
hypothetical protein Rv3822	Rv3822	30
Tuf	Rv0685	28
probable nonspecific lipid-transfer protein	Rv1627c	28
GroEL1	Rv3417c	28
LdtB	Rv2518c	27
PepD	Rv0983	24
PknH	Rv1266c	24

Ltp1	Rv2790c	23
PstS2	Rv0932c	22
TB22.2	Rv3036c	20
FadA3	Rv1074c	18
Mpt53	Rv2878c	18
LprA	Rv1270c	16
LprG	Rv1411c	16
PPE12	Rv0755c	14
EsxI	Rv1037c	1

Supplemental Table S4. Proteins selected on the basis of belonging to the protein family of a previously established protective antigen or on the basis of having a GO biological process related to the virulence or latency of MTB ranked, by number of promiscuous MHC-II epitopes.

**Name**	**Tuberculist ID**	**MHC-II Promiscuous Epitopes**
MmpL12	Rv1522c	263
PPE8	Rv0355c	194
PPE28	Rv1800	176
IleS	Rv1536	156
UvrA	Rv1638	116
FadD15	Rv2187	113
RpoB	Rv0667	109
PknD	Rv0931c	105
ClpB	Rv0384c	104
PE4	Rv0160c	100
Mce2A	Rv0589	100
PPE30	Rv1802	99

Mce1D	Rv0172	98
FadB	Rv0860	98
RecG	Rv2973c	98
FadD32	Rv3801c	97
probable aldehyde dehydrogenase	Rv0458	96
UvrB	Rv1633	92
HtpG	Rv2299c	89
PonA2	Rv3682	88
FtsH	Rv3610c	87
FadE5	Rv0244c	86
PE_PGRS11	Rv0754	86
FadD30	Rv0404	85
Mce2D	Rv0592	84
halimadienyl diphosphate synthase	Rv3377c	84
GyrB	Rv0005	83

PbpB	Rv2163c	79
EccA1	Rv3868	79
EccCa1	Rv3870	79
PonA1	Rv0050	77
Mce4A	Rv3499c	77
PbpA	Rv0016c	76
RecA	Rv2737c	76
FadD13	Rv3089	76
Mce1A	Rv0169	75
GroEL1	Rv3417c	74
ClpX	Rv2457c	73
Mce2C	Rv0591	72
Mce3D	Rv1969	72
FadD19	Rv3515c	72
Mce3C	Rv1968	71

CysN	Rv1286	70
Tgs4	Rv3088	70
EccB1	Rv3869	68
possible penicillin-binding lipoprotein	Rv2864c	66
Mce4F	Rv3494c	66
probable succinate dehydrogenase	Rv0248c	65
FadA2	Rv0243	62
Mpa	Rv2115c	62
LigD	Rv0938	61
HtrA	Rv1223	57
Mce4C	Rv3497c	57
Mce4D	Rv3496c	56
Icl1	Rv0467	55
Mce1C	Rv0171	54
Mce1F	Rv0174	53

Mce3A	Rv1966	53
PE26	Rv2519	53
SdhA	Rv3318	52
AccD4	Rv3799c	49
Mce2F	Rv0594	46
probable nonspecific lipid-transfer protein	Rv1627c	43
LdtB	Rv2518c	42
FbpC (Ag85C)	Rv0129c	40
TB22.2	Rv3036c	40
PknH	Rv1266c	39
GroEL2	Rv0440	36
PPE12	Rv0755c	36
Mpt53	Rv2878c	36
hypothetical protein Rv3822	Rv3822	36
LprQ	Rv0483	35

PepD	Rv0983	31
CaeA	Rv2224c	30
FadA3	Rv1074c	29
Tuf	Rv0685	23
PstS2	Rv0932c	23
LprG	Rv1411c	21
LprA	Rv1270c	17
Ltp1	Rv2790c	15
hypothetical protein Rv3811	Rv3811	7
EsxI	Rv1037c	4
